# Increased number of cases of giant cell arteritis and higher rates of ophthalmic involvement during the era of COVID-19

**DOI:** 10.1093/rap/rkaa067

**Published:** 2020-12-01

**Authors:** Rosamond Luther, Sarah Skeoch, John D Pauling, Christopher Curd, Felicity Woodgate, Sarah Tansley

**Affiliations:** 1 Royal National Hospital for Rheumatic Diseases and Royal United Hospitals Bath NHS Foundation Trust, Bath, UK; 2 Department of Pharmacy and Pharmacology, University of Bath, Bath, UK

**Keywords:** giant cell arteritis, vasculitis, behaviour, health policies, quality of health care, human activities

## Abstract

**Objectives:**

Our centre offers a fast-track assessment service for patients with suspected GCA and this service continued to operate during the coronavirus disease 2019 (COVID-19) pandemic. During and immediately following the peak of the COVID-19 pandemic in the UK we observed an increase in the number of patients diagnosed with GCA as well as an increased number of patients with visual complications. Our aim was to investigate this further.

**Methods:**

The electronic medical records of all patients referred for GCA fast-track assessment from January 2019 were reviewed. A complete list of patients undergoing temporal artery ultrasound and temporal artery biopsy for investigation of GCA dating back to 2015 was also available.

**Results:**

In the 12 week period between April and June 2020, 24 patients were diagnosed with GCA. Six (25%) had associated visual impairment. In contrast, during 2019, 28 new diagnoses of GCA were made in total and just 10% of patients suffered visual involvement. The number of patients diagnosed with GCA in April–June 2020 was nearly 5-fold that of the same time period the previous year. GCA diagnoses between April and June 2020 were supported by imaging (temporal artery ultrasound or CT-PET) in 72% of cases. We noted a higher proportion of male patients and a lower median age but no clear difference in the duration of symptoms prior to assessment.

**Conclusions:**

The reasons behind our observations remain unclear. However, our findings support the viral aetiopathogenesis hypothesis for GCA and demonstrate the importance of maintaining access to urgent rheumatology services during periods of healthcare disruption.

**Key messages**

- We observed an apparent increase in GCA cases following the UK COVID-19 pandemic peak.
- Services to rapidly assess an increased number of patients with GCA must be safeguarded.

## Introduction

GCA is a medical emergency that, if untreated, may lead to sight loss or stroke. Our rheumatology department established a GCA fast-track assessment pathway in 2015 and we aim to assess all patients within 2 working days of referral. All patients undergo temporal artery ultrasound, typically on the day of their rheumatologist assessment. Temporal artery biopsy is reserved for patients who have an unexpected positive or negative ultrasound result. Since the fast-track pathway was established, our trust has performed a median 34 temporal artery biopsies each year. A previous internal audit demonstrated that we diagnose ∼30 patients per year and that, in keeping with the literature, 10% of our cohort have ophthalmologist-confirmed visual impairment related to GCA [1].

The peak of the coronavirus disease 2019 (COVID-19) pandemic in the UK likely occurred around 8 April 2020 [2]. Redeployment of rheumatology staff at our trust occurred during the first 3 weeks in April 2020 in response to the COVID-19 pandemic and during this time the GCA pathway was overseen by two rheumatology consultants. From 22 April the pathway operated as usual and patients were assessed by the rheumatology registrar, supported by the on-call consultant. COVID-19-related restrictions on service delivery precluded temporal artery biopsy during this period, but temporal artery ultrasound availability was unaffected. During May–June 2020, in the immediate period following the UK’s COVID-19 pandemic peak, we observed a significant spike in the number of patients referred and diagnosed with GCA and an increased number of patients with visual complications. Here we report the characteristics of those patients diagnosed with GCA by our service between April and June 2020 compared with our previous experience.

## Methods

We retrospectively reviewed the medical records of patients referred to our fast-track pathway for possible GCA from January 2019 onwards. Prior to this, a large proportion of referrals were received via fax and unfortunately our referral records are incomplete. In order to ensure comparability with more recent 2020 data, the diagnosis recorded was that of the working diagnosis following initial patient assessment. A complete list of patients undergoing temporal artery ultrasound dating back to 2015 was available from the electronic records.

## Results

### Referral numbers and diagnoses

A total of 128 patients were referred to our fast-track assessment service in 2019, of whom 28 (22%) were diagnosed with GCA. Thus far in 2020 (data complete through the end of September) we have 118 referrals and 50 patients (42%) were diagnosed with GCA. Of these 50 new GCA diagnoses, 24 were made in the 12 week period between April and June 2020. This is close to the total number of 2019 new GCA diagnoses by our service in a 3 month period and is nearly five times the number of diagnoses made in the same time period in 2019.

**Fig. 1:**
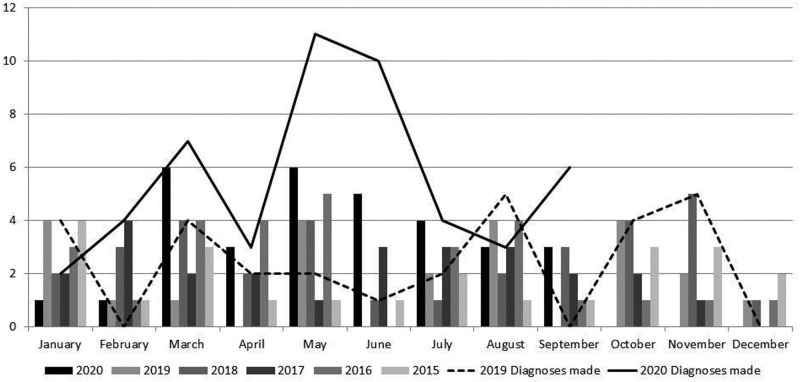
Number of patients with a positive temporal artery ultrasound scan each month over the last 5 years The number of patients diagnosed with GCA each month during 2019 and 2020 is also shown for comparison.

Since January 2015, our vascular studies department has performed a median 11 temporal artery ultrasound studies for possible GCA each month [interquartile range (IQR) 8.75–16]. Of these, a median of 19% (IQR 10–28) were reported as positive. In April 2020, 9 scans were undertaken, but in May and June this increased to 29 and 21 scans, respectively. The proportion of positive scans remained largely consistent with prior experience (21–33%). Following June, the number of GCA diagnoses dropped to more typical levels but remained at the higher end of what we had previously observed (see Fig. 1).

### Clinical features of patients diagnosed with GCA between April and June 2020

Of the patients diagnosed between April and June 2020, 25% had GCA-associated visual impairment, confirmed by an ophthalmologist examination. We also note that a higher proportion were male and the median age was lower (see Table 1).

**Table 1:** Characteristics of patients diagnosed with GCA in April–June 2020 compared with the previous year

Characteristics	April 2020	May 2020	June 2020	April– June 2019	January 2019– March 2020 (inclusive)
GCA diagnoses^a^, *n*	3	11	10	5	42^c^
Mean GCA diagnoses/week	0.75	2.75	2.5	0.4	0.7
Proportion of referrals with GCA diagnosis, %	33	39	52	17	20
Female, *n* (%)	1 (33)	8 (73)	3 (30)	4 (80)	30 (71)
Age, years, median (IQR)	66 (62.5–69)	75 (71.5–80.5)	77 (70–85)	81 (74–82)	74 (68–82)
Duration of symptoms prior to assessment, days, median (IQR)	7 (6.5–21)	28 (21–24.5)	7 (6–12)	21 (19–21)	21 (12–34)
Reviewed within 48 h of referral, *n* (%)	3 (100)	11(100)	9 (90)	3 (60)	27 (64)
Diagnoses confirmed with imaging or biopsy^b^, *n* (%)	2 (60)	8 (72)	7 (70)	2 (40)	25 (60)
Visual involvement, *n* (%)	1 (33)	2 (18)	3 (30)	1 (20)	4 (10)
Received i.v. methylprednisolone, *n* (%)	0 (0)	4 (36)	3 (30)	0 (0)	4 (10)
Received tocilizumab, *n* (%)	1 (33)	1(9)	1 (10)	0	3 (9)

a Working diagnosis following initial assessment as part of fast-track pathway.

b Imaging: temporal artery ultrasound, PET-CT or temporal artery biopsy. Temporal artery biopsy unavailable due to COVID-19 restrictions on services from April 2020.

c Includes the five cases referred to in the preceding columns April–June 2019.

During this time period GCA assessment was arranged within 48 h of referral for all patients except one, whom we had difficulty contacting. The diagnosis of GCA was made on clinical grounds following a full assessment: 62% of patients had characteristic temporal artery ultrasound findings and a further 10% had PET-CT changes, suggesting large vessel vasculitis. All patients with visual symptoms were examined by an ophthalmologist. There were no notable differences in referral patterns: with one exception, all referrals were received from general practitioners located within the usual catchment area of our trust. The remaining patient was referred by the acute medical team following hospital admission with a lacunar infarct and anterior ischaemic optic neuropathy secondary to GCA.

## Discussion

The wider healthcare implications of the COVID-19 pandemic are potentially far-reaching. Reports of altered healthcare-seeking behaviour due to perceptions of personal risk and concerns regarding overburdening the health service have, for many conditions, reduced healthcare utilization [3]. A 75% reduction in utilization of GCA fast-track pathways since the COVID-19 outbreak in Italy compared with the previous year has been reported and this was accompanied by an increase in the proportion of patients with GCA-associated visual loss [4]. In contrast to this previous report, we have seen an increase in both the number GCA diagnoses and associated visual loss during the initial wave of COVID-19 in our UK hospital. While a greater proportion of our referrals were diagnosed with GCA than in 2019, the higher proportion of diagnoses supported by imaging suggests clinical diagnoses were not being made more readily owing to restrictions around access to diagnostic temporal artery biopsies. Indeed, due to concerns that steroid use may be a risk factor for vulnerability to COVID-19, as a department we felt that a diagnosis of GCA and the associated steroid exposure would be well justified. Some patients almost certainly delayed seeking medical attention due to concerns regarding COVID-19, and this assumption fits with the median duration of symptoms prior to assessment being highest in May, although the overall duration of symptoms for April–June 2020 remains similar to the previous year. Previous studies have linked delayed GCA treatment to an increased risk of visual complications [5, 6].

Despite the worldwide pandemic, we are the first to report this pattern, however, the incidence of GCA is known to be highest in northern European countries compared with the Mediterranean, Middle East and Asia [1]. Additional work is required to establish whether our local experiences represent a broader increase in GCA diagnoses across the UK. If confirmed, potential drivers may include psychological stress [7], shifts in previously identified seasonality [8] or COVID-19 itself. Previous work has examined the potential role of viruses such as varicella zoster proposed as potential triggers in GCA [9]. None of our patients with GCA had symptoms suggesting COVID-19 infection, despite detailed questioning as part of steroid counselling (we were not testing asymptomatic patients during this period). However, asymptomatic COVID-19 infection has been widely reported, including in older adults [10]. An immune-mediated post-infectious syndrome, paediatric multisystem inflammatory syndrome temporally associated with severe acute respiratory syndrome coronavirus 2 (PIMS-TS), which is related to asymptomatic or mildly symptomatic COVID-19 infection has also been reported in children [11] and there are parallels between the immunopathogenesis of GCA, including the role of IL-6, and current understanding about the aetiopathogenesis of PIMS-TS [12]. No patients with GCA developed symptoms of COVID-19 on follow-up assessment and thus far a limited number of tested patients have not had SARS-CoV-2 antibodies, suggesting previous COVID-19 exposure.

## Conclusion

We observed a large increase in the number of patients referred and diagnosed with GCA in May and June 2020, the period following the peak of the COVID-19 pandemic in the UK. Importantly, a higher proportion of patients also suffered associated visual impairment. While the reasons behind this are as yet unclear, our findings support the viral aetiopathogenesis hypothesis for GCA and highlight the importance of maintaining urgent access to rheumatology services during this time of uncertainly and reorganization of healthcare services.

*Funding*: No specific funding was received from any funding bodies in the public, commercial or not-for-profit sectors to carry out the work described in this article.

*Disclosure statement*: The authors have declared no conflicts of interest.

## Data availability statement

The data underlying this article will be shared on reasonable request to the corresponding author.
